# Non-diabetes status after diagnosis of impaired glucose tolerance and risk of long-term death and vascular complications: A post hoc analysis of the Da Qing Diabetes Prevention Outcome Study

**DOI:** 10.1371/journal.pmed.1004419

**Published:** 2024-07-09

**Authors:** Xin Qian, Jinping Wang, Qiuhong Gong, Yali An, Xinxing Feng, Siyao He, Xiaoping Chen, Wenjuan Wang, Lihong Zhang, Yuanchi Hui, Xiuwei Zhai, Bo Zhang, Yanyan Chen, Guangwei Li

**Affiliations:** 1 Endocrinology Centre, Fuwai Hospital, Chinese Academy of Medical Sciences and Peking Union Medical College, Beijing, China; 2 Department of Cardiology, Da Qing First Hospital, Da Qing, China; 3 Department of Endocrinology, China-Japan Friendship Hospital, Beijing, China; 4 Division of Non-Communicable Disease Control and Community Health, Chinese Center for Disease Control and Prevention, Beijing, China; 5 Department of Neurosurgery, Da Qing First Hospital, Da Qing, China

## Abstract

**Background:**

The association between years of non-diabetes status after diagnosis of impaired glucose tolerance (IGT) and the risk of long-term death and cardiovascular outcomes needed to be clarified.

**Methods and findings:**

In this post hoc analysis, we included 540 individuals with IGT who participated in the original Da Qing Diabetes Prevention Study (DQDPS). In the DQDPS, all participants were diagnosed with IGT by a 75 g oral glucose tolerance test and randomized to intervention or control groups with a 6-year lifestyle intervention trial. After the completion of the trial, death, cardiovascular events, and microvascular complications were monitored over a 30-year follow-up. In this post hoc analysis, the Cox analysis assessed the extended risk of these outcomes in individuals who either remained non-diabetes status or progressed to diabetes at the end of 2, 4, and 6 years after diagnosis of IGT. In all participants, the difference in the cumulative incidence rate of the outcomes between the diabetes and non-diabetes group gradually increased over 30 years. Compared with the diabetes group, a significantly lower risk of all-cause death (hazard ratio [HR]: 0.74; 95% confidence interval [CI]: 0.57 to 0.97, *p* = 0.026), cardiovascular events (HR: 0.63; 95% CI: 0.49 to 0.82, *p* < 0.001), and microvascular complications (HR: 0.62; 95% CI: 0.45 to 0.86, *p* = 0.004) first emerged in individuals who remained non-diabetes at the 4 years visit, whereas the significant risk reduction in cardiovascular death was first observed at the end of 6 years (HR: 0.56; 95% CI: 0.39 to 0.81, *p* = 0.002) after adjustment for age, sex, smoking status, BMI, systolic blood pressure, blood glucose, total cholesterol, intervention, and medications (including insulin plus oral hypoglycaemics, antihypertensives, and lipid-lowering agents). The results in the original intervention group alone were similar to the whole group. The main limitations of our study are the limited number of participants and the sole ethnicity of the Chinese population.

**Conclusions:**

In this study, we observed that maintaining several years of non-diabetes status after IGT diagnosis was associated with a significant reduction in long-term risk of death and vascular complications, and for most of these outcomes, maintaining at least 4 years of non-diabetes status may be needed to achieve a significant risk reduction.

## Abstract 2

Xin Qian and co-authors explore how the duration of a lifestyle intervention for diabetes prevention influences length of time spent in non-diabetes status after a diagnosis of impaired glucose tolerance and, the impact on long term outcomes.

## Author summary

**Why was this study done?:**

- Lifestyle intervention can reduce or delay the incidence of diabetes in people with impaired glucose tolerance (IGT).
- Major lifestyle intervention trials have shown that lifestyle intervention can improve microvascular complications and some cardiovascular risk factors.
- To the best of our knowledge, no study has reported the relationship between long-term outcomes and duration of maintaining non-diabetes status after a diagnosis of IGT which may be influenced by the duration of lifestyle intervention, after IGT diagnosis.

**What did the researchers do and find?:**

- We conducted a post hoc analysis of the China Da Qing Diabetes Prevention Study (DQDPS), a 6-year lifestyle intervention trial in people with IGT.
- In this study, we included 540 individuals with IGT and assessed the long-term risk of mortality, macro- and microvascular diseases between those who remained non-diabetes status or not at the end of 2, 4, and 6 years visit after diagnosis of IGT.
- Our analyses showed that individuals who remained non-diabetic for at least 4 years after being diagnosed with IGT had a significantly lower risk of all-cause death, cardiovascular events, and microvascular complications compared with those who progressed to diabetes. However, this effect was not observed in individuals who remained non-diabetic for a shorter period.

**What do these findings mean?:**

- This post hoc analysis suggests that a longer duration of non-diabetes status in those with IGT has beneficial health outcomes and reduces mortality.
- The implementation of effective interventions targeting those with IGT should be considered as part of preventative management for diabetes and diabetes-related vascular complications.
- The main limitations of this study are the limited number of participants and the sole ethnicity of the Chinese population.

## Introduction

Type 2 diabetes is associated with an increased risk of death, disability, and fatal vascular complications and poses a significant economic burden on individuals and societies worldwide [1], especially in China [2]. Researchers have made extensive efforts to prevent diabetes and obtained strong evidence and important achievements that have changed our knowledge of whether diabetes can be prevented. Among individuals with impaired glucose tolerance (IGT), lifestyle interventions (diet and/or exercise) are feasible and effective in delaying and decreasing the incidence of diabetes [3] and are more effective than metformin in high-risk populations [4].

The 3 “landmark” diabetes prevention studies reported long-term follow-up outcomes in individuals with IGT after lifestyle or metformin intervention. The Diabetes Prevention Program Outcomes Study (DPPOS) found that during a 21-year follow-up period, regression to normal glucose tolerance (NGT) was associated with a lower prevalence of microvascular diseases [5], whereas neither metformin nor lifestyle intervention reduced cardiovascular events [6]. The Finnish Diabetes Prevention Study (DPS) found no reduction in cardiovascular morbidity in individuals with IGT during the first 10-year follow-up [7]. The Da Qing Diabetes Prevention Study (DQDPS) and Outcomes Study (DQDPOS) found that lifestyle modifications were associated with all-cause and cardiovascular disease (CVD)-related deaths [8]. A recent study reported that the incidence of all-cause death, cardiovascular death, CVD events, and composite microvascular complications in a lifestyle intervention group was significantly lower than that in controls [9].

The American Diabetes Association released the “Prevention or Delay of Type 2 Diabetes and Associated Comorbidities: Standards of Care in Diabetes—2023” [10], which reported that lifestyle intervention in individuals with a high risk of type 2 diabetes affected the risk of CVD [11] and reduced the risk of long-term microvascular in the DPPOS and DQDPS [12,13]. The effect of lifestyle interventions on the prevention of CVD and death has also been demonstrated in the DQDPOS [8,9]. However, these clinical practice recommendations do not specify the target number of years for delaying the onset of diabetes through lifestyle interventions to prevent the associated comorbidities.

The present analysis focused on exploring the association between maintaining a non-diabetes status (remaining IGT or regression to NGT) after IGT diagnosis and the long-term risk of death and diabetes-related vascular complications. To our knowledge, this is the first study that investigates the association between the diabetes-free years after diagnosis of IGT and the risk of long-term death and cardiovascular outcomes. It may provide evidence to support the target number of years for delaying the onset of diabetes through lifestyle interventions to prevent associated comorbidities.

## Methods

### Study design and participants

In this post hoc analysis, all 540 participants with IGT were recruited from the original DQDPS. At the beginning of the DQDPS, among the 110,660 residents of Da Qing City in 1986, all participants were assessed for glucose tolerance status based on the results of a standard 75 g oral glucose tolerance test (OGTT) according to the 1985 World Health Organization diabetes criteria [14]. From 1986 to 1992, 576 individuals diagnosed with IGT in 1986 participated in a lifestyle intervention trial consisting of 438 participants in the intervention group and 138 in the control group [3]. During this period, OGTTs were performed to systematically assess diabetes status at the end of 2, 4, and 6 years after diagnosis of IGT. These 3 years were referred to as the 3 key point years. In between those times, diabetes was diagnosed by physicians during regular clinical visits. In the intervention group, lifestyle intervention was continued for another 2 years in individuals who remained non-diabetes status (remaining IGT or regression to NGT) at a key point year of the trial until they progressed to diabetes or reached the end of 6 years. The intervention was discontinued in individuals who progressed to diabetes at any time. If individuals progressed to diabetes at any stage of the trial, regardless of whether they were assigned to the intervention or non-intervention group, they were instructed to receive routine treatment for diabetes at their local health facilities according to the Chinese Diabetes Treatment Guidelines. Follow-up studies to trace clinical outcomes were conducted at 20, 23, 30, and 34 years after enrolment. During the over 30-year follow-up study, 36/576 (6.3%) participants were lost to follow-up, and 31/36 (86%) were lost during the first 6 years (1986 to 1992).

### Ethics statements

This study was approved by the Ethics Committee of Fuwai Hospital (approval number: 2020–1390). All participants or the representatives of the deceased participants provided written informed consent.

### Outcomes

The definitions of the outcomes were consistent with those of previous reports [9,15]. CVD events included coronary heart disease, hospitalization for heart failure, and non-fatal or fatal strokes. Coronary heart disease was defined as non-fatal or fatal myocardial infarction or sudden death. Composite severe microvascular disease was the aggregate outcome of retinopathy, nephropathy, and neuropathy. Retinopathy was defined as a history of photocoagulation, blindness due to retinal disease, or proliferative retinopathy. Nephropathy was defined as a history of end-stage renal disease, renal dialysis, renal transplantation, or death due to chronic kidney disease. Neuropathy was defined as a history of leg, ankle, or foot ulceration; gangrene; or amputation. Cardiovascular death was defined as death due to myocardial infarction, sudden death, heart failure, or stroke. Medical records and death certificates were reviewed to determine causes of death. For each outcome, onset was defined as the earliest date of recognition of the outcome. This information was first derived from interviews using standardized questionnaires and then verified by medical records and/or death certificates. Two physicians who were blinded to the participants’ trial assignments independently adjudicated the outcomes.

### Statistics analyses

In this post hoc analysis, a three-step analysis was performed to investigate the association between years of maintaining a non-diabetes status (remaining IGT or regression to NGT) during the trial and the risk of clinical outcomes. To explore the minimum years of maintaining non-diabetes status after diagnosis of IGT related to the long-term risk of outcomes, we compared the data between the non-diabetes and diabetes groups which were identified at the 3 prespecified key point visits of the original DQDPS and referred them to as Steps 1, 2, and 3, respectively. The first key point year was at the end of 2 years after diagnosis of IGT (1986 to 1988); the second one was at the end of 4 years (1986 to 1990); the third one was at the end of 6 years (1986 to 1992). In Step 2, the analysis included the participants in Step 1, and in Step 3, the analysis included the participants in Steps 1 and 2. The reason for these “three key points” was that the prespecified systemic OGTT, which aimed to assess diabetes status, was repeated at these years during the 6-year trial. The three-step analyses were individually performed to determine the earliest period at which a significant risk reduction in outcomes was observed between the non-diabetes and diabetes groups. To further explore the influence of maintaining a non-diabetes status on the long-term risk of outcomes considering the lifestyle intervention, the same analysis was completed in the original intervention group alone.

Missing data of baseline characteristics were imputed multiple times to provide appropriate estimates within participants (Table 1). The average baseline values, such as blood lipid levels, were calculated using the available data. Baseline characteristics are expressed as means ± standard deviations or interquartile ranges (25th to 75th percentiles) for continuous characteristics and numbers and percentages (%) for categorical characteristics. For comparisons, the *t* test was used to analyze data with a normal distribution, the Wilcoxon rank-sum test was used to analyze data with a right-skewed distribution, and the chi-squared test was used to analyze categorical variables. Cox model analysis was performed to evaluate the hazard ratios (HRs) and 95% confidence intervals (CIs) for each outcome between the non-diabetes and diabetes groups. The timescales of the outcomes were assessed from the 3 key point years (1988, 1990, and 1992) to the end of follow-up (2020), with 32-, 30-, and 28-year follow-up times, respectively. The potential confounders were age, sex, smoking status, BMI, blood pressure, total cholesterol levels, blood glucose, medications, and intervention. The medications used for hypoglycaemia, hypertension, and dyslipidaemia, were summed up over the 30-year follow-ups. The cumulative incidence of all outcomes for the non-diabetes and diabetes groups at each key point year was demonstrated using Kaplan–Meier curves.

**Table 1:** Baseline characteristics of all participants based on diabetes status at the end of 3 key point years after diagnosis of IGT.

	At the end of 2 years		At the end of 4 years		At the end of 6 years	
	Non-diabetes (*n* = 469)	Diabetes (*n* = 70)	Non-diabetes (*n* = 357)	Diabetes (*n* = 176)	Non-diabetes (*n* = 266)	Diabetes (*n* = 254)
**Age, years**	45.2 ± 9.0	45.3 ± 9.9	45.1 ± 9.1	45.1 ± 9.1	44.7 ± 9.1	45.0 ± 8.7
**Male, *n* (%)**	251 (54)	46 (66)	198 (56)	95 (54)	148 (56)	135 (53)
**Smoker, *n* (%)**	190 (41)	28 (40)	152 (43)	63 (36)	114 (43)	92 (36)
**BMI, kg/m** ^**2**^	25.8 ± 3.8	26.4 ± 4.7	25.4 ± 3.7	26.8 ± 4.3^†^	25.3 ± 3.8	26.7 ± 4.0^†^
**Systolic blood pressure, mmHg**	133 ± 23	130 ± 35	131 ± 22	135 ± 29	130 ± 22	135 ± 27*
**Fasting plasma glucose, mmol/L**	5.5 ± 0.8	5.9 ± 0.9^†^	5.5 ± 0.8	5.8 ± 0.8^†^	5.4 ± 0.8	5.8 ± 0.8^†^
**1-h plasma glucose, mmol/L**	11.1 ± 2.2	12.2 ± 2.6^†^	11.0 ± 2.2	11.8 ± 2.2^†^	10.6 ± 2.2	11.9 ± 2.2^†^
**2-h plasma glucose, mmol/L**	8.9 ± 0.9	9.4 ± 0.9^†^	8.9 ± 0.9	9.2 ± 0.9^†^	8.8 ± 0.9	9.2 ± 0.9^†^
**Total cholesterol, mmol/L**	5.1 ± 1.3	5.0 ± 1.0	5.0 ± 1.3	5.3 ± 1.4*	4.9 ± 1.3	5.2 ± 1.3*
**Intervention randomization, *n* (%)**	365 (78)	40 (57)^†^	289 (81)	111 (63)^†^	220 (83)	170 (67)
**Medications over 30 years**						
Insulin plus oral hypoglycaemics, *n* (%)	250 (53)	44 (63)	184 (52)	110 (63)*	129 (49)	165 (65)^†^
Antihypertension, *n* (%)	234 (50)	35 (49)	176 (49)	91 (52)	131 (49)	136 (54)
Lowering lipid, *n* (%)	207 (44)	39 (54)	162 (45)	79(45)	130 (49)	111 (44)

*p < 0.05.

^†^*p* < 0.01.

IGT, impaired glucose tolerance; at the end of 2 years: during the period from 1986 to 1988; at the end of 4 years: during the period from 1986 to 1990; at the end of 6 years: during the period from 1986 to 1992; non-diabetes included individuals remaining IGT or regression to normal glucose tolerance; diabetes included individuals who progressed to diabetes. The diabetes status based on the data at the end of 2, 4, and 6 years. Treatment medications were summed up over the 30-year follow-up.

Statistical significance was set at a two-sided *p*-value of less than 0.05. Data analyses were performed using SAS version 9.4 (SAS Institute, Cary, North Carolina, United States of America) and Stata 16.0 SE (Stata Corp., College Station, Texas, USA).

## Results

At the end of 1988, 539 participants remained alive, with 70 having diabetes and 469 remaining non-diabetes. At the end of 1990, 176 of the 533 participants had been diagnosed with diabetes and 357 had not. At the end of 1992, 520 patients were alive, including 254 who progressed to diabetes and 266 who did not (Table 1).

Compared with individuals remaining non-diabetes status for 2 years, those who were identified as having diabetes during the same period had higher glucose levels (*p* < 0.01). Compared with individuals remaining non-diabetes status for 4 years, those with diabetes had a higher BMI, blood pressure, plasma glucose levels, and percentage of smokers (*p* < 0.05). Compared with individuals remaining non-diabetes status for 6 years, those with diabetes had higher total cholesterol levels and more frequent use of insulin or oral hypoglycaemics (*p* < 0.05) and a higher BMI, blood pressure, glucose levels, and percentage of smokers (*p* < 0.05) (Table 1). Over the 30-year follow-up period, 54%, 49%, and 44% of the patients received medications for the treatment of hyperglycaemia, hypertension, and dyslipidaemia, respectively.

**Fig 1:**
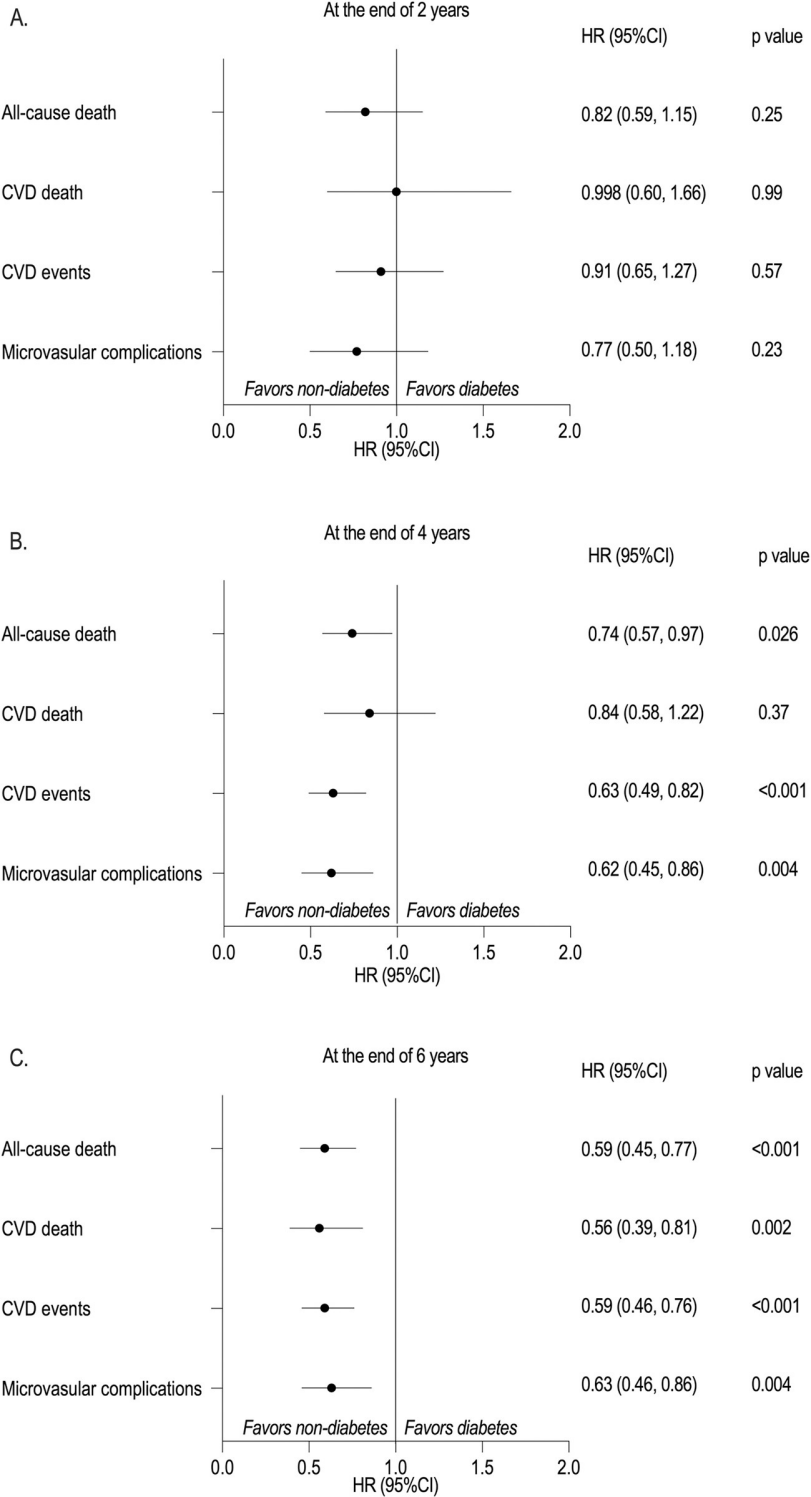
Relationship between the years of maintaining non-diabetes status after diagnosis of IGT and long-term risk of outcomes in all participants. (A) At the end of 2 years, (B) at the end of 4 years, and (C) at the end of 6 years. IGT, impaired glucose tolerance; CVD, cardiovascular disease. At the end of 2 years: during the period from 1986 to 1988; at the end of 4 years: during the period from 1986 to 1990; at the end of 6 years: during the period from 1986 to 1992; non-diabetes included individuals remaining IGT or regression to normal glucose tolerance; diabetes included individuals who progressed to diabetes. HRs were calculated after adjustment for age, sex, smoking status, BMI, systolic blood pressure, total cholesterol, blood glucose, intervention, and medications (including insulin plus oral hypoglycaemics, antihypertensives, and lipid-lowering agents).

Table 2 shows the influence of the years of maintaining a non-diabetes status after diagnosis of IGT on death and vascular complications in all participants. At the end of the key point of 2 years, there was no significant difference in death and vascular complication rates per 1,000 person-years between those who remained non-diabetes for 2 years and those who progressed to diabetes during the same period (*p* > 0.05). HRs (non-diabetes versus diabetes) for all-cause and CVD death were 0.82 (95% CI: 0.59 to 1.15, *p* = 0.25) and 0.998 (95% CI: 0.60 to 1.66, *p* = 0.99), respectively, and macro- and microvascular complications were 0.91 (95% CI: 0.65 to 1.27, *p* = 0.57) and 0.77 (95% CI: 0.50 to 1.18, *p* = 0.23), respectively (Fig 1A).

**Table 2:** Influence of the years of maintaining non-diabetes status after diagnosis of IGT on death and vascular complications in the entire group.

	Number of events		Event rate/1,000 person-year (95% CI)			Non-diabetes vs. diabetes		Non-diabetes vs. diabetes	
	Non-diabetes	Diabetes	Non-diabetes	Diabetes	*p*-value	Crude HR (95% CI)	*p*-value	Adjusted HR (95% CI)	*p*-value
**At the end of 2 years**									
All-cause death	256	44	22.1 (19.6, 25.0)	26.5 (19.7, 35.6)	0.27	0.81 (0.59, 1.12)	0.21	0.82 (0.59, 1.15)	0.25
CVD death	128	19	11.1 (9.3, 13.2)	11.4 (7.3, 17.9)	0.87	0.94 (0.58, 1.53)	0.81	0.998 (0.60, 1.66)	0.99
CVD events	260	46	27.4 (24.3, 31.0)	34.6 (25.9, 46.2)	0.15	0.77 (0.56, 1.05)	0.1	0.91 (0.65, 1.27)	0.57
Microvascular complications	161	28	15.1 (12.9, 17.6)	18.9 (13.1, 27.4)	0.27	0.70 (0.47, 1.04)	0.08	0.77 (0.50, 1.18)	0.23
**At the end of 4 years**									
All-cause death	184	110	22.4 (19.4, 25.8)	28.0 (23.3, 33.8)	0.063	0.78 (0.62, 0.99)	0.04	0.74 (0.57, 0.97)	0.026
CVD death	92	53	11.2 (9.1, 13.7)	13.5 (10.3, 17.7)	0.27	0.82 (0.58, 1.14)	0.24	0.84 (0.58, 1.22)	0.37
CVD events	177	122	26.2 (22.6, 30.3)	40.7 (34.1, 48.6)	<0.001	0.61 (0.49, 0.77)	<0.001	0.63 (0.49, 0.82)	<0.001
Microvascular complications	114	75	14.9 (12.4, 17.9)	21.9 (17.5, 27.5)	0.01	0.59 (0.44, 0.79)	<0.001	0.62 (0.45, 0.86)	0.004
**At the end of 6 years**									
All-cause death	128	153	22.0 (18.5, 26.2)	28.9 (24.7, 33.9)	0.022	0.75 (0.59, 0.94)	0.014	0.59 (0.45, 0.77)	<0.001
CVD death	61	83	10.4 (8.2, 13.5)	15.7 (12.6, 19.4)	0.017	0.66 (0.47, 0.91)	0.013	0.56 (0.39, 0.81)	0.002
CVD events	125	167	26.2 (22.0, 31.3)	42.0 (36.1, 48.8)	<0.001	0.60 (0.48, 0.76)	<0.001	0.59 (0.46, 0.76)	<0.001
Microvascular complications	82	107	15.0 (12.1, 18.7)	23.4 (19.4, 28.3)	0.003	0.57 (0.43, 0.76)	<0.001	0.63 (0.46, 0.86)	0.004

Adjusted HR: adjustment for age, sex, smoking status, BMI, systolic blood pressure, blood glucose, total cholesterol, intervention, and medications (including insulin plus oral hypoglycaemics, antihypertensives, and lipid-lowering agents).

IGT, impaired glucose tolerance; CI, confidence interval; HR, hazard ratio; CVD events, cardiovascular disease events, first occurrence of fatal and non-fatal myocardial infarctions, fatal and non-fatal strokes, or hospitalized heart failure; CVD death, death due to myocardial infarction, sudden death, heart failure, or stroke; and microvascular complications, first occurrence of retinopathy, nephropathy, or neuropathy.

At the end of 2 years: during the period from 1986 to 1988; at the end of 4 years: during the period from 1986 to 1990; at the end of 6 years: during the period from 1986 to 1992; non-diabetes included individuals remaining IGT or regression to normal glucose tolerance; diabetes included individuals who progressed to diabetes.

At the end of key point of 4 years, the rates of vascular complications in individuals remaining non-diabetes status were lower than in those who progressed to diabetes. The rates per 1,000 person-year were 26.2 (22.6 to 30.3) versus 40.7 (34.1 to 48.6) for cardiovascular events and 14.9 (12.4 to 17.9) versus 21.9 (17.5 to 27.5) for microvascular disease (all *p* < 0.05), respectively. The long-term risk of all-cause death decreased by 26% among individuals remaining non-diabetes for 4 years, even after adjustment for age, sex, smoking status, BMI, blood pressure, total cholesterol levels, blood glucose, medications, and intervention (HR: 0.74; 95% CI: 0.57 to 0.97; *p* = 0.026). The risk of vascular complications decreased by 40% in these individuals (HR: 0.63; 95% CI: 0.49 to 0.82; *p* < 0.001 and HR: 0.62; 95% CI: 0.45 to 0.86; *p* = 0.004 for macro- and microvascular complications, respectively [Fig 1B]).

At the end of the key point of 6 years, the rates per 1,000 person-years for death and vascular complications were significantly different between individuals remaining non-diabetes status for 6 years and those who progressed to diabetes (all *p* < 0.05). Among these, a significant reduction in all-cause death and vascular complications persisted, as observed at the end of the key point of 4 years. The risk of cardiovascular death first decreased significantly by 44% (HR: 0.56; 95% CI: 0.39 to 0.81) after adjustment for confounders (*p* = 0.002) (Fig 1C). At the end of 6 years, the risk of each outcome was 40% lower in the non-diabetes group than in the diabetes group, even after adjusting for medications (all *p* < 0.01) (Table 2).

**Fig 2:**
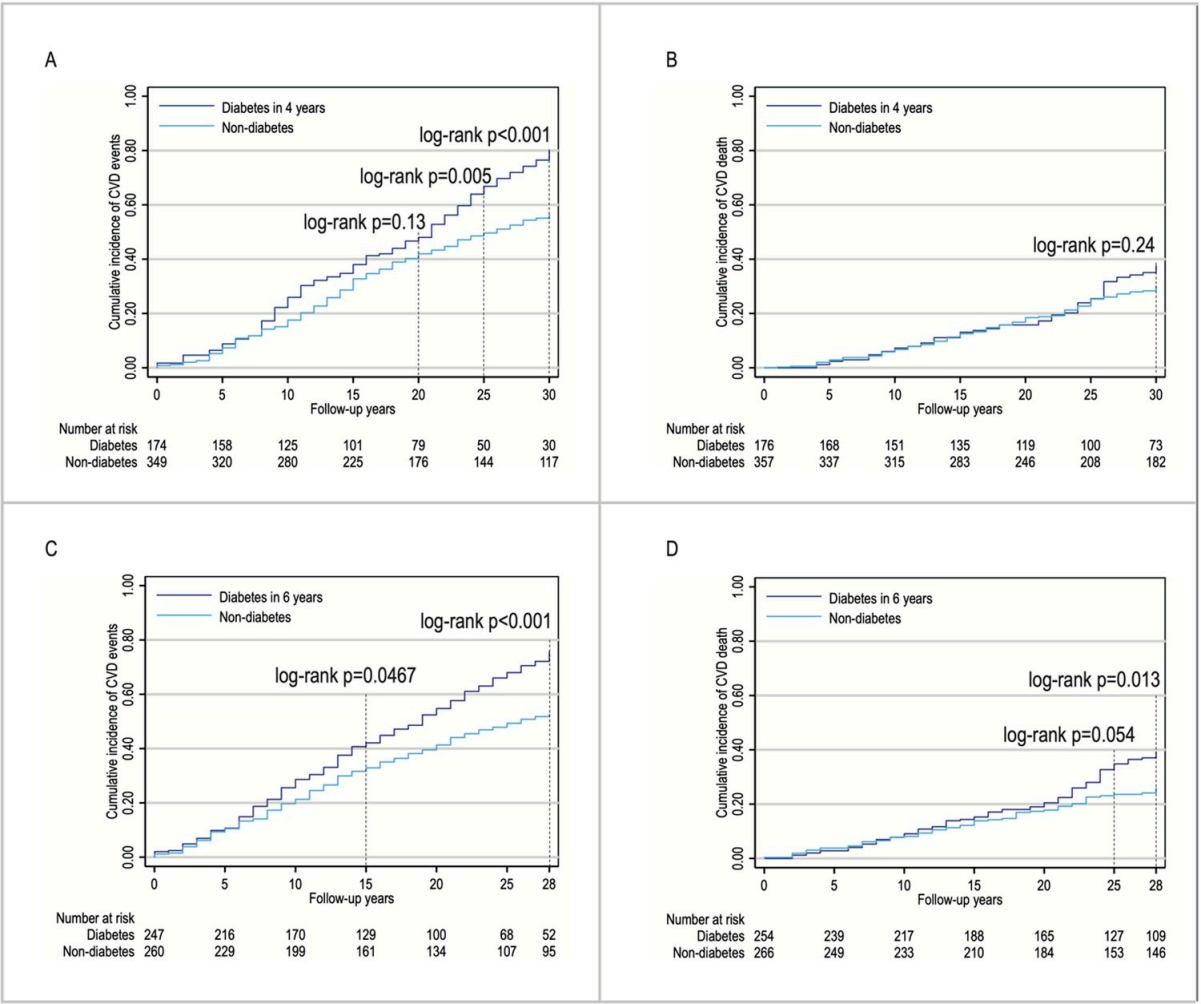
Kaplan–Meier failure curves of cardiovascular death and events at the end of key point of 4 and 6 years. (A) CVD events at the end of 4 years, (B) CVD death at the end of 4 years, (C) CVD events at the end of 6 years, and (D) CVD death at the end of 6 years. CVD, cardiovascular disease. At the end of 2 years: during the period from 1986 to 1988; at the end of 4 years: during the period from 1986 to 1990; at the end of 6 years: during the period from 1986 to 1992; non-diabetes included individuals remaining IGT or regression to normal glucose tolerance; diabetes included individuals who progressed to diabetes.

Fig 2 shows the log-rank *p*-values of the cumulative incidence of CVD events and death between the non-diabetes and diabetes groups at the end of 4 years (Fig 2A and 2B) and at the end of 6 years (Fig 2C and 2D) at different follow-up years. At the end of 4 years, the log-rank *p*-values for CVD events were 0.13 and 0.005 at 20 and 25 years of follow-up, respectively (Fig 2A). At the end of 6 years, the log-rank *p*-value for CVD events was 0.0467 over 15 years of follow-up (Fig 2C). For cardiovascular death, the log-rank *p*-value was less than 0.05 only at the end of 6 years over the 28-year follow-up period (Fig 2B and 2D).

Table 3 shows the influence of the years of maintaining a non-diabetes status after diagnosis of IGT on death and vascular complications in the original intervention group. The results were consistent with those in the whole study population. At the end of 2 years, the risk of each outcome reduced; however, the changes were not statistically significant (*p* > 0.05). At the end of 4 years, the risk of all-cause death (HR: 0.71; 95% CI: 0.52 to 0.96; *p* = 0.026) and vascular complications (HR; 0.59; 95% CI: 0.43 to 0.80; *p* = 0.001 and HR: 0.61; 95% CI: 0.42 to 0.90; *p* = 0.012 for macro- and microvascular disease, respectively) was lower in individuals who remained non-diabetes status than in those who progressed to diabetes. These differences persisted at the end of 6 years. At the end of 6 years, the risk of cardiovascular death reduced by 50% (HR: 0.52; 95% CI: 0.33 to 0.80; *p* = 0.003). The baseline clinical characteristics are shown in S1 Table.

**Table 3:** Influence of the years of maintaining non-diabetes status after diagnosis of IGT on death and vascular complications in the original intervention group.

	Number of events		Event rate/1,000 person-year (95% CI)			Non-diabetes vs. diabetes		Non-diabetes vs. diabetes	
	Non-diabetes	Diabetes	Non-diabetes	Diabetes	*p-*value	Crude HR (95% CI)	*p*-value	Adjusted HR (95% CI)	*p-*value
**At the end of 2 years**									
All-cause death	193	24	21.1 (18.3, 24.3)	25.5 (17.1, 38.1)	0.38	0.81 (0.53, 1.23)	0.32	0.70 (0.45, 1.08)	0.11
CVD death	93	11	10.2 (8.3, 12.5)	11.7 (6.5, 21.1)	0.64	0.84 (0.45, 1.57)	0.59	0.73 (0.39, 1.40)	0.35
CVD events	197	24	26.3 (22.8, 30.2)	32.2 (21.6, 48.1)	0.34	0.80 (0.52, 1.22)	0.29	0.71 (0.46, 1.10)	0.12
Microvascular complications	126	13	14.9 (12.5, 17.7)	15.1 (8.8, 26.1)	0.93	0.90 (0.51, 1.59)	0.72	0.85 (0.47, 1.56)	0.61
**At the end of 4 years**									
All-cause death	145	67	21.5 (18.2, 25.2)	26.5 (20.8, 33.6)	0.16	0.80 (0.60, 1.07)	0.14	0.71 (0.52, 0.96)	0.026
CVD death	68	34	10.1 (7.9, 12.8)	13.4 (9.6, 18.8)	0.17	0.74 (0.49, 1.12)	0.15	0.76 (0.49, 1.18)	0.23
CVD events	141	74	25.5 (21.6, 30.0)	38.6 (30.7, 48.4)	0.005	0.64 (0.48, 0.84)	0.002	0.59 (0.43, 0.80)	0.001
Microvascular complications	93	46	14.8 (12.1, 18.1)	20.7 (15.5, 27.6)	0.066	0.63 (0.44, 0.90)	0.011	0.61 (0.42, 0.90)	0.012
**At the end of 6 years**									
All-cause death	103	99	21.1 (17.4, 25.7)	27.2 (22.4, 33.2)	0.074	0.76 (0.58, 1.00)	0.055	0.61 (0.45, 0.83)	0.002
CVD death	44	57	9.0 (6.7, 12.1)	15.7 (12.1, 20.3)	0.006	0.57 (0.38, 0.84)	0.005	0.52 (0.33, 0.80)	0.003
CVD events	99	112	24.8 (20.4, 30.2)	41.5 (34.5, 50.0)	<0.001	0.80 (0.44, 0.76)	<0.001	0.59 (0.43, 0.80)	0.001
Microvascular complications	65	74	14.2 (11.1, 18.1)	23.5 (18.7, 29.6)	0.003	0.52 (0.37, 0.73)	<0.001	0.56 (0.39, 0.81)	0.002

Adjusted HR: adjustment for age, sex, smoking status, BMI, systolic blood pressure, blood glucose, total cholesterol, and medications (including insulin plus oral hypoglycaemics, antihypertensives, and lipid-lowering agents).

IGT, impaired glucose tolerance; CI, confidence interval; HR, hazard ratio; CVD events, cardiovascular disease events, first occurrence of fatal and non-fatal myocardial infarctions, fatal and non-fatal strokes, or hospitalized heart failure; CVD death, death due to myocardial infarction, sudden death, heart failure, or stroke; and microvascular complications, first occurrence of retinopathy, nephropathy, or neuropathy.

At the end of 2 years: during the period from 1986 to 1988; at the end of 4 years: during the period from 1986 to 1990; at the end of 6 years: during the period from 1986 to 1992; non-diabetes included individuals remaining IGT or regression to normal glucose tolerance; diabetes included individuals who progressed to diabetes.

## Discussion

The post hoc analysis demonstrated that the risk of long-term outcomes in participants with IGT who remained non-diabetes status for at least 4 years decreased gradually and significantly compared with those who progressed to diabetes during the same period, if individuals were followed up for a sufficiently long period. Over 28 years of follow-up, a significant reduction in the risk of cardiovascular death was found in individuals remained non-diabetes status for 6 years after being diagnosed with IGT. Similar results were founded in the original intervention group alone, maintaining 6 years of non-diabetes status was needed to reduce the long-term risk of outcomes, including cardiovascular mortality, significantly.

To the best of our knowledge, this post hoc analysis is the first to report the relationship between the years of maintaining a non-diabetes status after diagnosis of IGT and the risk of long-term outcomes. The question of how many years of remaining non-diabetes status after diagnosis of IGT are needed to reduce their long-term CVD risk has continuously plagued diabetologists. The present analysis demonstrated that the 30-year risks of macro- and microvascular complications, and all-cause death were reduced by 37%, 38%, and 26% in individuals remaining non-diabetes status at the end of 4 years visit during the trial, respectively, compared with those who progressed to diabetes during the same period. A significant reduction in the risk of cardiovascular death was observed in individuals remaining non-diabetes at the end of 6 years visit. These results suggest that in high-risk diabetic populations, a “4-year threshold” existed in the duration of maintaining a non-diabetes status and exceeding this “threshold” may reduce the risk of death and vascular complications. Therefore, the present study reminds us that in Chinese adults, the prevention of diabetes by lifestyle modification in individuals with IGT requires the maintenance of non-diabetes status for at least 4 years if it aims to reduce the long-term subsequent risk of vascular complications and all-cause death. For cardiovascular death, no less than 6 years of maintaining a non-diabetes status after the diagnosis of IGT is needed.

As previous trials have proved that intervention can delay and reduce diabetes, the intervention influenced the duration of progression from IGT to diabetes during which the intervention trial’s length plays a vital role. Compared with the DPP and DPS, the DQDPS had the longest lifestyle prevention trial (median: 6 [DQDPS] versus 3.5 [DPP] and 4 [DPS] years) as it recorded the OGTT results every 2 years (from the beginning to the end of the trial, 0 to 6 years) and the non-diabetes status period in detail. The long-term follow-up period was over 30 years in the DQDPS. These data made it possible to investigate the association between years of maintaining non-diabetes status after diagnosis of IGT and the long-term risk of outcomes. In this post hoc analysis, a significant reduction in the long-term risk of death and vascular complications occurred in individuals who remained non-diabetes status for 4 or 6 years after being diagnosed with IGT during the trial. This result indicates that to reduce long-term adverse outcomes, maintaining several years of a non-diabetes status is needed in individuals with IGT. This study aimed to address the question of how non-diabetes status diagnosed at various stages of a lifestyle intervention trial (i.e., the time from entry) may be significantly associated with a reduced risk of subsequent adverse clinical outcomes, such as cardiovascular events and death. It may potentially inform the design of future diabetes prevention studies targeted at reducing risk of progression to diabetes and improving risk of long-term adverse clinical outcomes.

Although extremely important, the years of maintaining a non-diabetes status after diagnosis of IGT is only one of the most important factors influencing the risk of long-term outcomes. The mechanisms underlying the different long-term outcomes are complex. The DPPOS discussed the influence of post-trial overuse of medication for the treatment of hypertension, hyperglycaemia, and dyslipidaemia on outcomes, which may mask the beneficial influence of lifestyle interventions on these outcomes [16]. The present post hoc analysis revealed that the reduction in the risk of vascular complications remained significant after adjusting for medications. Additionally, compared with participants in the DPPOS and DPS, those in the DQDPS were younger (45.2 ± 9.3 [DQDPS] versus 50.5 ± 11 [DPPOS] and 55 ± 7 [DPS] years), had a lower percentage of female (45.8% [DQDPS] versus 68.5% [DPPOS] and 67.1% [DPS]), and had a lower BMI (25.7 ± 7.6 [DQDPS] versus 34 ± 6.7 [DPPOS] and 31.5 ± 4.5 [DPS] kg/m^2^) [17]. These differences in the ethnicity of the studied populations and the intensity of the intervention may have contributed to the outcomes. Nevertheless, the difference in the duration of lifestyle interventions among the 3 diabetes prevention studies remains one of the most important differences [3,4,18]. Therefore, one possible explanation is that the different durations of the interventions across the three studies may have affected the years of maintaining a non-diabetes status, which is associated with the long-term risk of death and vascular complications. An intervention that lasts for several years is needed to observe its apparent effects on death and vascular complications; however, it is difficult to establish a control group and implement long-term follow-up.

We recently reported that about 90% of the participants in DQDPS progressed to diabetes during the 20 years follow-up period. Furthermore, the China Diabetes Prevention Program (CDPP) reports that currently Chinese with prediabetes face a high risk of developing diabetes and the initial combination of metformin and lifestyle intervention modification (LSM) showed a better effect of preventing diabetes than LSM alone [19]. Therefore, it may be logical to assume that the combined use of metformin and LSM may be more effective in prolonging diabetes free time.

The question arises as to why remaining several years of non-diabetes status after diagnosis of IGT can affect clinical outcomes over decades of follow-up. Studies on diabetes have demonstrated that strict, early, glycaemic control can improve long-term adverse outcomes through a phenomenon called the “legacy” effect. The 10-year post-trial follow-up of the United Kingdom Prospective Diabetes Study showed that the risk of myocardial infarction and all-cause death was continuously lower in both intensive management groups compared with the standard management group in participants with newly diagnosed type 2 diabetes [20]. This demonstrates that early improvements in glycaemic control have a legacy effect, which reduces the risk of coronary events and death from any cause. A similar legacy effect has been demonstrated in 3 studies on diabetes prevention. These studies showed that the effect of a 3- to 6-year lifestyle intervention on diabetes prevention extended to a post-trial follow-up period of more than 10 years [21–23]. Our previous study reported that the median delay in diabetes duration in individuals who remained non-diabetes status for 6 years was 17 years [15]. Hence, it is easy to understand why time-limiting lifestyle interventions decrease the subsequent long-term risk of adverse clinical outcomes.

Follow-up time is another important factor in determining the positive association between vascular complications and death and maintaining non-diabetes time. Compared with the DPPOS (21-year follow-up) and DPS (13-year follow-up), the DQDPS had a follow-up time of more than 30 years. In the DQDPS, a significant reduction in the long-term risk of vascular complications and death did not occur in the same follow-up period as DPPOS, but gradually. The incidence in microvascular complications [13], death (cardiovascular and all-cause) [8], and all micro- and macrovascular complications and death [9] was significantly reduced in the 20-, 23-, and 30-year follow-up studies, respectively. Furthermore, in a 30-year follow-up study, individuals who still had IGT at the end of a 6-year trial had significantly lower incidences of death and vascular complications than those who developed diabetes [15]. This shows that a short follow-up period may not elucidate the “real” result of vascular outcomes, which needs a decades-long post-trial follow-up. Our post hoc analysis explored the combined influence of maintaining non-diabetes time (4 to 6 years) and post-trial follow-up (>30 years) on death and macrovascular complications. To prevent diabetes among the Chinese IGT population, maintaining a non-diabetes status for a minimum of 4 years combined with 25 years of post-trial follow-up may be needed to reduce the risk of macrovascular complications and death significantly. Concerning Western populations, which usually have a higher BMI than Asian populations, the number of years of maintaining a non-diabetes status after diagnosis of IGT are required to reduce the risk of these clinical outcomes remains under investigation.

The original lifestyle intervention group’s results were similar to those of the entire study population. The HRs (non-diabetes versus diabetes group) for all outcomes at the end of 2, 4, and 6 years in the original intervention group were lower than those in the entire group. Although the underlying mechanism is unclear, the difference in long-term diabetes status transformation between individuals with or without intervention may be involved. To some degree, the intervention-induced longer maintenance of non-diabetes status may contribute to the association with the risk reduction.

This post hoc analysis has several strengths. First, the data used in this study are from the world’s first randomized lifestyle intervention study on the prevention of diabetes, and a previous report proved that lifestyle intervention in individuals with IGT could effectively delay the onset of diabetes during the trial and for more than 10 years after trial completion. Second, the cohort was followed up for more than 30 years and revealed that time-limited lifestyle interventions could reduce the long-term cumulative incidence of all-cause death, cardiovascular death, and vascular complications [24]. Third, the study had the longest intervention time and longest duration of post-trial follow-up among other similar diabetes intervention trials worldwide.

The limitations of this post hoc analysis are as follows. First, it included a limited number of participants. Second, this study was conducted solely in a Chinese population. In view of the demographic and cultural differences, and variations in obesity, smoking status, education, and access to health care, a similar investigation in another population is warranted. Third, we cannot exclude the influence of other unmeasured confounders which could explain the association between duration of remaining diabetes free after IGT diagnosis and outcomes. Fourth, due to the original DQDPS design (IGT-people who progressed to diabetes and with severe hyperglycaemia were not suitable candidates to receive a 75 g glucose load OGTT), some participants did not join the regular interview at the end of 2, 4, and 6 years; therefore, the effects of the changes of clinical characteristics during these time points could not be evaluated.

## Conclusions

In conclusion, this post hoc analysis found that the risk reduction of death and long-term vascular complications in people with IGT is significantly associated with the duration of remaining non-diabetes after an IGT diagnosis. This suggests that a longer diabetes-free time may lower the risk of long-term adverse outcomes. Taking action, including but not limited to lifestyle intervention, to prolong the diabetes-free time in people with prediabetes may be crucial.

## Supporting information

S1 STROBE Checklist Checklist of items that should be included in reports of cohort studies. (DOCX)

S1 Table Baseline characteristics of participants in the intervention group alone based on diabetes status at 3 key point years after diagnosis of IGT. (DOCX)

## Abbreviations

CI: confidence interval
CVD: cardiovascular disease
DPPOS: Diabetes Prevention Program Outcomes Study
DPS: Diabetes Prevention Study
DQDPS: Da Qing Diabetes Prevention Study
HR: hazard ratio; IGT, impaired glucose tolerance
LSM: lifestyle intervention modification
NGT: normal glucose tolerance
OGTT: oral glucose tolerance test
