# Supplementary material for: Non-diabetes status after diagnosis of impaired glucose tolerance and risk of long-term death and vascular complications: A post hoc analysis of the Da Qing Diabetes Prevention Outcome Study
Source: PLoS Med. 2024 Jul 9;21(7):e1004419. doi: 10.1371/journal.pmed.1004419 (PMC11233008; doi:10.1371/journal.pmed.1004419)
Supplement: S1 Table — (DOCX) [file pmed.1004419.s002.docx]

**S1 Table.** Baseline characteristics of participants in the original intervention group alone based on diabetes status at three-key point years after diagnosis of IGT.

|  | **At the end of 2 years** | | **At the end of 4 years** | | **At the end of 6 years** | |
| --- | --- | --- | --- | --- | --- | --- |
|  | Non-diabetes (n=365) | Diabetes (n=40) | Non-diabetes (n=289) | Diabetes (n=111) | Non-diabetes (n=220) | Diabetes (n=170) |
| **Age, years** | 44.9±9.0 | 43.3±8.7 | 44.9±9.1 | 43.8±8.5 | 44.2±9.1 | 44.5±8.3 |
| **Male sex, n (%)** | 198 (54) | 23 (58) | 160 (55) | 58 (52) | 120 (55) | 91 (54) |
| **Smoker, n (%)** | 138 (38) | 15 (38) | 119 (41) | 32 (29)^*^ | 91 (41) | 54 (32) |
| **BMI, kg/m^2^** | 25.8±3.8 | 25.8±5.3 | 25.4±3.8 | 26.7±4.4^†^ | 25.3±3.9 | 26.6±4.0^†^ |
| **Systolic blood pressure, mmHg** | 133±23 | 131±33 | 131±22 | 137±28^*^ | 129±22 | 136±27^†^ |
| **Fasting plasma glucose, mmol/L** | 5.6±0.8 | 6.1±0.8^†^ | 5.5±0.8 | 5.9±0.8^†^ | 5.5±0.8 | 5.8±0.8^†^ |
| **1-h plasma glucose, mmol/L** | 11.1±2.1 | 12.1±3.0^†^ | 11.0±2.2 | 11.8±2.3^†^ | 10.6±2.1 | 12.0±2.3^†^ |
| **2-h plasma glucose, mmol/L** | 8.9±0.9 | 9.5±0.9^†^ | 8.9±0.9 | 9.2±0.9^†^ | 8.9±0.9 | 9.1±0.9^†^ |
| **Total cholesterol, mmol/L** | 5.0±1.4 | 5.1±1.1 | 4.9±1.3 | 5.3±1.6 | 4.9±1.3 | 5.2±1.4^*^ |
| **Medications over 30 years** |  |  |  |  |  |  |
| Insulin plus oral hypoglycaemics, n (%) | 196 (54) | 24 (60) | 150 (52) | 70 (63) | 107 (49) | 113 (67)^†^ |
| Antihypertension, n (%) | 188 (52) | 20 (50) | 147 (51) | 61 (55) | 108 (49) | 100 (59) |
| Lowering lipid, n (%) | 167 (46) | 21 (53) | 135 (47) | 53 (48) | 108 (49) | 80 (48) |

^*^p<0.05, ^†^p<0.01

IGT, impaired glucose tolerance; at the end of 2 years: during the period from 1986 to 1988; at the end of 4 years: during the period from 1986 to 1990; at the end of 6 years: during the period from 1986 to 1992; non-diabetes included individuals remaining IGT or regression to normal glucose tolerance; diabetes included individuals who progressed to diabetes. The diabetes status based on the data at the end of 2, 4, and 6 years. Treatment medications were summed up over the 30-year follow-up.
